# Isorhamnetin and Quercetin Derivatives as Anti-Acetylcholinesterase Principles of Marigold (*Calendula officinalis*) Flowers and Preparations

**DOI:** 10.3390/ijms18081685

**Published:** 2017-08-02

**Authors:** Daniil N. Olennikov, Nina I. Kashchenko, Nadezhda K. Chirikova, Anzurat Akobirshoeva, Ifrat N. Zilfikarov, Cecile Vennos

**Affiliations:** 1 Institute of General and Experimental Biology, Siberian Division, Russian Academy of Science, Sakh’yanovoy Street 6, Ulan-Ude 670047, Russia; ninkk@mail.ru; 2 Department of Biochemistry and Biotechnology, North-Eastern Federal University, 58 Belinsky Street, Yakutsk 677027, Russia; hofnung@mail.ru; 3 Pamir Biological Institute, Kholdorova Street 1, Khorog 736002, Tajikistan; akobirshoeva-a@mail.ru; 4 All-Russian Institute of Medical and Aromatic Plants, Greena Street 7/1, Moscow 117216, Russia; zilfikarovin@mail.ru; 5 Regulatory and Medical Scientific Affairs, Padma AG, 1 Underfeldstrasse, CH-8340 Hinwil, Switzerland; vennos_c@mail.ru

**Keywords:** *Calendula officinalis*, marigold, acetylcholinesterase inhibition, quercetin, isorhamnetin, HPLC

## Abstract

Marigold (*Calendula officinalis* L.) is one of the most common and widespread plants used medicinally all over the world. The present study aimed to evaluate the anti-acetylcholinesterase activity of marigold flowers, detect the compounds responsible and perform chemical analysis of marigold commercial products. Analysis of 23 varieties of *C. officinalis* flowers introduced into Siberia allowed us to select the Greenheart Orange variety due to the superior content of flavonoids (46.87 mg/g) and the highest inhibitory activity against acetylcholinesterase (IC_50_ 63.52 µg/mL). Flavonoids, isorhamnetin and quercetin derivatives were revealed as potential inhibitors with the application of high-performance liquid chromatography (HPLC) activity-based profiling. Investigation of the inhibitory activity of isorhamnetin glycosides demonstrated the maximal potency for isorhamnetin-3-*O*-(2′′,6′′-di-acetyl)-glucoside (IC_50_ 51.26 μM) and minimal potency for typhaneoside (isorhamnetin-3-*O*-(2′′,6′′-di-rhamnosyl)-glucoside; IC_50_ 94.92 µM). Among quercetin derivatives, the most active compound was quercetin-3-*O*-(2′′,6′′-di-acetyl)-glucoside (IC_50_ 36.47 µM), and the least active component was manghaslin (quercetin-3-*O*-(2′′,6′′-di-rhamnosyl)-glucoside; IC_50_ 94.92 µM). Some structure-activity relationships were discussed. Analysis of commercial marigold formulations revealed a reduced flavonoid content (from 7.18–19.85 mg/g) compared with introduced varieties. Liquid extract was the most enriched preparation, characterized by 3.10 mg/mL of total flavonoid content, and infusion was the least enriched formulation (0.41 mg/mL). The presented results suggest that isorhamnetin and quercetin and its glycosides can be considered as potential anti-acetylcholinesterase agents.

## 1. Introduction

Cognitive-mental deficiency is one of the key signs of the disturbances of higher nervous activity in many disease states, including neurodegenerative diseases such as Alzheimer’s disease (AD), Parkinson’s disease, neuroinfections and others. The clinical picture of cognitive mnestic deficits manifests in disturbance of concentration, a decrease in motivation and malfunction of abstract and logical thinking, as well as different versions of memory disorders [1]. In some cases, this pathology progresses steadily and leads to severe disability of patients; in other cases, it is a slow progressive process, and therefore, the recovery of patients’ working capacity stretches on for months or even years. In this regard, effective correction of cognitive and mnestic disorders is not only a medical, but also a social problem. The creation of new drugs that contribute to the restoration of thinking and memory is an urgent direction of investigations in the field of neuropharmacology.

The modern strategy of developing drugs for the correction of cognitive mnestic impairments is based on the molecular mechanisms of brain function and the nature of their violation in specific pathological states [2]. At the same time, mnestic deficits of any origin have a number of common pathogenic reasons and mechanisms of development. In this regard, changes in cholinergic transmission among biochemical disorders occupy a special place. These changes are associated with the loss of cholinergic neurons, malfunction of acetylcholine production or its release and downregulation of cholinergic receptors. The currently available information indicates that the regulation of cholinergic neurotransmission is an effective tool for reducing the side effects caused by the expression of mnestic deficits. In this way, the prescription of acetylcholinesterase inhibitors is one of the possible pharmacological approaches for the treatment of these disorders [3,4].

Inhibition of acetylcholinesterase is the most effective therapeutic approach to the restoration of the cholinergic system in patients with cognitive mnestic impairments. Some herbal medicines such as rivastigmine or galantamine, which inhibit acetylcholinesterase may be applied for the treatment of early stages of AD since these compounds increase the level of endogenous acetylcholine and thus enhance cholinergic neurotransmission [1].

According to the literature data, the availability of anti-cholinesterase activity has been demonstrated for more than 300 natural compounds such as: alkaloids (53%), monoterpenes (10%), coumarins (7%), triterpenes (6.5%), flavonoids (5%), simple phenols (5%) and others [5]. Despite the efficacy of alkaloid and terpene compounds in the process of acetylcholinesterase inhibition, their wide application in medical therapy is limited due to the presence of toxic properties. In contrast, natural low molecular phenolic compounds (LMPC) such as flavonoids, xanthones and phenylpropanoids do not possess this side effect and can be used in long-term therapy of diseases. The data on the impact of LMPC on acetylcholinesterase activity are insufficient; moreover, there are no data on advanced structural-functional investigations and the structural features causing the presence of the anti-cholinesterase activity of these compounds. These circumstances suggest that the investigation of LMPC of natural origin for the presence of anti-cholinesterase activity is of great practical interest.

Early investigations of *Calendula officinalis* have shown that extracts of this plant species have an inhibitory effect on acetyl- and butyrylcholinesterase [6]. Methanol extract from the flowers of *C. officinalis* revealed the most pronounced activity. Determination of the compounds responsible for this activity was not carried out, and therefore, the mechanism of the inhibitory effect of *C. officinalis* on cholinesterase is unclear.

It should be noted that *C. officinalis* extracts exhibit a wide spectrum of biological activity on the central and peripheral nervous system. In particular, the protective effect of *C. officinalis* extracts against neurotoxic oxidative stress induced by monosodium glutamate (MSG) and excitotoxic brain damage was previously shown [7]. Treatment with the extract significantly attenuated behavioral alterations, oxidative stress and hippocampal damage in MSG-treated animals. *C. officinalis* extract exhibited analgetic activity on a model of an acetic acid-induced writhing test [8]. The application of the extract in doses of 100–300 mg/kg significantly increased the tail flick latency. The aqueous ethanol extract from *C. officinalis* flowers showed both spasmogenic and spasmolytic effects through calcium channel blocking and cholinergic activity [9]. High doses of *C. officinalis* extract may have sedative effects and increase sleep time [10]. The data about low acute and subchronic toxicity of *C. officinalis* extracts [11] allows us to consider that *C. officinalis* extract is a prospective neuropharmacological remedy for the treatment of a wide range of diseases.

The present research is aimed at chemical examination of 23 varieties of *C. officinalis* flowers introduced into Siberia and determination of their acetylcholinesterase inhibiting activity, detection of the most active compounds responsible for the manifestation of anti-acetylcholinesterase activity with the use of high-performance liquid chromatography (HPLC) activity-based profiling and revealing the active compound content in marigold flower commercial samples.

## 2. Results and Discussion

### 2.1. Chemical Composition and Anti-Acetylcholinesterase Potential of 23 Varieties of C. officinalis Flowers

Based on known data of the chemical composition of *C. officinalis* flowers, we investigated the most evident correlations between the parameters of compound content and the values of anti-acetylcholinesterase inhibition. For this purpose, the total extracts of flowers of 23 varieties of *C. officinalis* introduced into Siberia were analyzed to determine the content of essential oil, carotenoids, triterpenoids, flavonoids, phenylpropanoids and polysaccharides, as well as the index of 50% inhibition of acetylcholinesterase in in vitro experiments (Table 1).

The total essential oil content in the varieties analyzed was from 0.32 (Cardinal) to 3.04 mg/g (Jiga-Jiga) dry extract weight. Variations of carotenoid and triterpene content were 2.63 (Touch of Red) to 11.39 mg/g (Rose Surprise) and 10.28 (Flame Dancer) to 65.70 mg/g (Egypt Sun), respectively. The basic phenolic groups of total extracts of *C. officinalis* flowers were flavonoids and phenylpropanoids with content values of 10.52 (Jiga-Jiga) to 46.87 mg/g (Greenheart Orange) and 6.07 (Golden Prince) to 33.47 mg/g (Golden Imperator), respectively. The concentration of polysaccharide components in *C. officinalis* flowers extracts varied from 11.09 (Rose Surprise) to 44.15 mg/g (Honey Cardinal).

Available data about the quantitative chemical composition of *C. officinalis* describes the content of essential oil, carotenoids, triterpenoids and flavonoids. Essential oil as a minor component is present in *C. officinalis* flowers at values of 1.0 mg/g (Brazil) [12], 1.0–2.7 mg/g (Egypt) [13] and 1.3–9.7 mg/g (South Africa) [14]. The carotenoid concentration in *C. officinalis* flowers may vary in a wide range: 0.25–2.17 mg/g (Italy) [15]), 0.4–2.76 mg/g (Romania) [16], 1.0–1.7 mg/g (Japan) [17], 2.0–35.1 mg/g (Estonia) [18]. The triterpenoid content of *C. officinalis* flowers may reach levels of 20 mg/g (Germany) [19], 20.53 mg/g (Poland) [20] or 25.98–40.82 mg/g (Italy) [21]. Previously declared data about the content of flavonoids in *C. officinalis* flowers collected in different places were 2.1–6.8 mg/g (Estonia) [22], 2.5–8.8 mg/g (Bratislava) [23], 6.3–7.9 mg/g (Brazil) [24] and 18.3–36.3 mg/g (Italy) [15]. This demonstrates the good ability of the Siberian cultivars of *C. officinalis* to concentrate the bioactive components in flowers.

**Figure 1:**
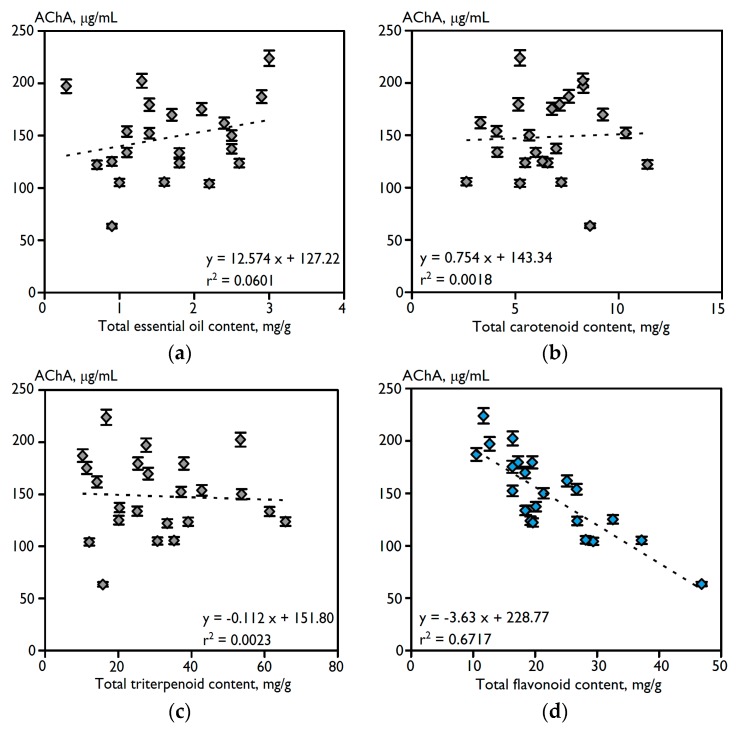

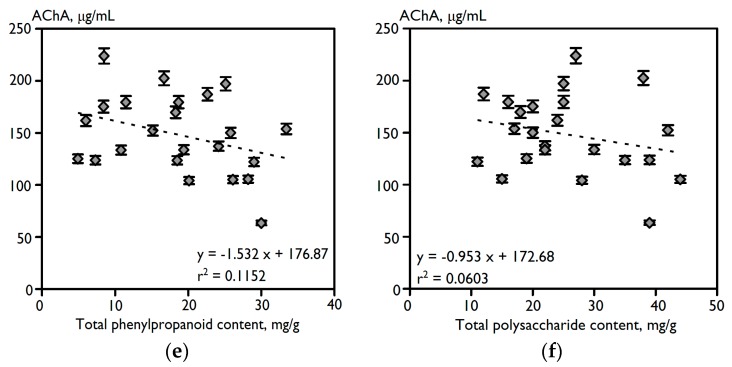
Correlation graphs (dashed lines) between total content of essential oil (**a**), carotenoids (**b**), triterpenoids (**c**), flavonoids (**d**), phenylpropanoids (**e**) and polysaccharides (**f**) (mg/g) in total extracts of flowers of 23 varieties of *C. officinalis* and their anti-acetylcholinesterase activity value (AChA; IC_50_, μg/mL). Reference compounds: neostigmine (positive control; active, IC_50_ = 0.75 ± 0.01 μg/mL), NaCl (negative control; inactive).

The range of acetylcholinesterase inhibitory value (IC_50_) of total extracts of 23 varieties of *C. officinalis* flowers was from 223.9 μg/mL for the least effective sample, the Jiga-Jiga variety, to 63.5 μg/mL for the most active sample, the Greenheart Orange variety. The inhibitory activity of a Turkish sample of *C. officinalis* was lower, reaching 22.37% at a dose of 1000 μg/mL for methanolic extract [6]. To understand the correlation among all of the studied chemical parameters and biological potential, linear regression analysis was used (Figure 1). The highest correlation was observed between total flavonoid content and anti-acetylcholinesterase activity (*r*^2^ = 0.6717). No other class of phytocomponents demonstrated appropriate correlations due to the low *r*^2^ value: essential oil (0.0601), carotenoids (0.0018), triterpenoids (0.0023), phenylpropanoids (0.1152) and polysaccharides (0.0603). Previously, flavonoids were demonstrated to have good correlative dependency with the anti-acetylcholinesterase activity of natural extracts of *Smallanthus sonchifolius* [25], propolis [26] and *Garcinia parvifolia* [27].

### 2.2. Flavonoid Profile of C. officinalis Flowers’ Extract and High-Performance Liquid Chromatography (HPLC) Activity-Based Profiling of Acetylcholinesterase Inhibitors

**Figure 2:**
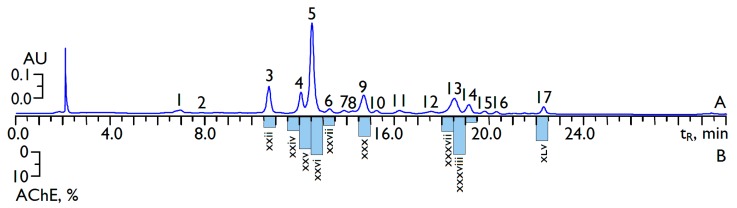
Chromatograms (microcolumn reversed-phase high-performance liquid chromatography with ultraviolet detection (MC-RP-HPLC-UV)) of 60% ethanol extract from *C. officinalis* of the Greenheart Orange variety at 350 nm (A) and HPLC-based anti-acetylcholinesterase activity (AChE) profiling (B). The bar graphs on B show the inhibitory activity of the individual HPLC fractions collected from a single separation. Compounds: 1, 3-*O*-сaffeoylquinic acid; 2, caffeic acid; 3, manghaslin; 4, calendoflavobioside; 5, typhaneoside; 6, rutin; 7, isoquercitrin; 8, quercetin-3-*O*-(2′′-ramnosyl)-rhamnoside; 9, calendoflavoside; 10, 3,5-di-*O*-caffeoylquinic acid; 11, quercetin-3-*O*-(6′′-acetyl)-glucoside; 12, 1,5-di-*O*-caffeoylquinic acid; 13, narcissin; 14, isorhamnetin-3-*O*-glucoside; 15, calendoflaside; 16, 4,5-di-*O*-caffeoylquinic acid; 17, isorhamnetin-3-*O*-(6′′-acetyl)-glucoside. Rome numbers indicate numbers of fractions after semi-preparative HPLC microfractionation. AU—absorbance units.

According to the preliminary stage of the study, the Greenheart Orange variety of *C. officinalis* flowers was selected and investigated as the most active sample with anti-acetylcholinesterase activity and superior flavonoid content. In order to examine the phenolic profile of the selected marigold, its 60% ethanol extract from flowers was subjected to a previously developed microcolumn reversed-phase HPLC procedure with ultraviolet (UV) detection (MC-RP-HPLC-UV) [28]. From the comparison of retention times, UV and mass spectrometry (MS) data with reference substances, 12 flavonoids and five phenylpropanoids were detected (Figure 2, Table 2).

The flavonoids were both quercetin (3,5,7,3′,4′-pentahydroxyflavone) and isorhamnetin (3,5,7,4′-tetrahydroxy-3′-methoxyflavone) derivatives, all in the form of glycosides. The sugar components were various combinations of glucose and rhamnose such as 3-*O*-glucosides (isoquercitrin, Peak 7; isorhamnetin-3-*O*-glucoside, Peak 14), 3-*O*-(6′′-acetyl)-glucosides (Peaks 11 and 17), 3-*O*-(2′′-rhamnosyl)-rhamnosides (Peaks 8 and 15), 3-*O*-(2′′-rhamnosyl)-glucosides or 3-*O*-neohesperidosides (calendoflavobioside, Peak 4; calendoflavoside, Peak 9), 3-*O*-(6′′-rhamnosyl)-glucosides or 3-*O*-rutinosides (rutin, Peak 6; narcissin, Peak 13) and 3-*O*-(2′′,6′′-di-rhamnosyl)-glucosides (manghaslin, Peak 3; typhaneoside, Peak 5). The non-flavonoid components were phenylpropanoids such as 3-*O*-caffeoylquinic acid (Peak 1), caffeic acid (Peak 2) and three di-*O*-caffeoylquinic acids (Peaks 10, 12 and 16) (Figure S1).

The flavonoids isoquercitrin, isorhamnetin-3-*O*-glucoside, calendoflavobioside, calendoflavoside, rutin, narcissin and isorhamnetin-3-*O*-(2′′-rhamnosyl)-rhamnosides (calendoflaside) had been previously reported in *C. officinalis* [29], as well as manghaslin and typhaneoside [30]. Quercetin-3-*O*-(6′′-acetyl)-glucoside, isorhamnetin-3-*O*-(6′′-acetyl)-glucoside, quercetin-3-*O*-(2′′-rhamnosyl)-rhamnosides and the mentioned phenylpropanoids were identified as components of seven Russian varieties of *C. officinalis* [28]. The total flavonoid content in *C. officinalis* extract was 92.54 mg/g, consisting of 26.75 mg/g of quercetin derivatives and 65.79 mg/g of isorhamnetin derivatives (Table 2). Typhaneoside (42.46 mg/g), narcissin (12.92 mg/g), manghaslin (12.62 mg/g) and calendoflavobioside (10.12 mg/g) were the prevailing flavonoid compounds. The concentration of phenylpropanoids was not more 10 mg/g of dry weight of extract.

To identify the compounds of interest in *C. officinalis* flower extract with high acetylcholinesterase inhibitory activity, the extract investigated was submitted to HPLC activity-based profiling. This technique is a miniaturized and highly effective approach for localization and characterization of bioactive natural products with minute amounts of injected extracts [31,32,33]. This technique combines the speed and separation power of HPLC with the structural information of online spectroscopy and miniaturized bioassays. For the detection of acetylcholinesterase inhibitors in *C. officinalis* flower extract, the procedure of small-scale semi-preparative microfractionation by reversed-phase HPLC was used. This yielded 60 microfractions of 30 s each that were transferred to a deep-well microtiter plate. Then, microfractions were dried, redissolved in buffer solution and subjected to post-chromatographic reaction with an acetylcholinesterase/α-naphthyl acetate/Fast Blue B salt model system to evaluate the anti-acetylcholinesterase activity. The anti-acetylcholinesterase activity of the microfractions after post-column derivatization is shown in Figure 2B. Major inhibition was observed in Fractions xxv, xxvi, xxx, xxxviii and xiv, which displayed the highest anti-cholinesterase activity potential with inhibition values of 14.2%, 16.1%, 8.5%, 16.9% and 9.8%, respectively, while the activity of the other fractions was not significantly different from zero. The data obtained showed considerable acetylcholinesterase inhibiting activity of the fractions containing flavonoids. The majority of compounds eluted in the most active fractions were derivatives of isorhamnetin like typhaneoside (Fraction xxvi), calendoflavoside (Fraction xxx), narcissin (Fraction xxxviii) and isorhamnetin-3-*O*-(6′′-acetyl)-glucoside (Fraction xlv), while only calendoflavobioside was a derivative of quercetin (Fraction xxv). The fractions containing other derivatives of quercetin are characterized as inhibitors of moderate power, and caffeoylquinic acids do not show a pronounced mode of action.

### 2.3. Acetylcholinesterase Inhibitory Activity of C. officinalis Flavonoids

**Figure 3:**
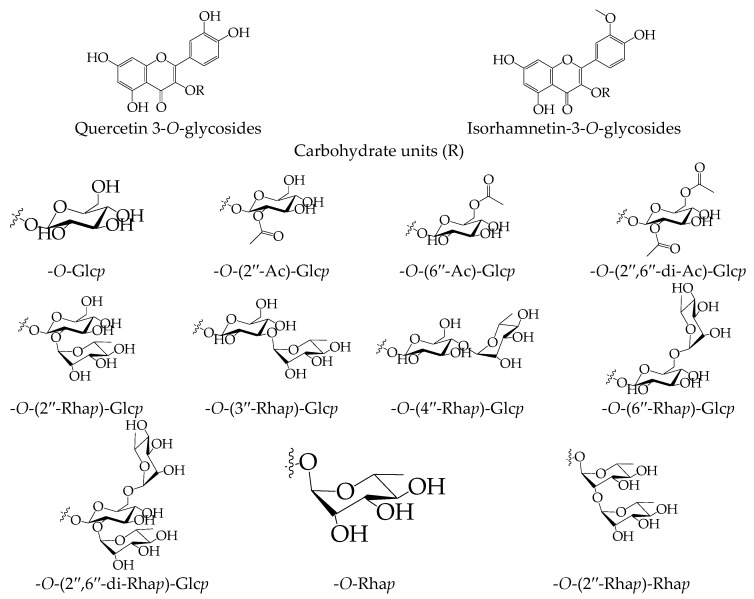
Structures of quercetin and isorhamnetin glycosides from *C. officinalis*. Abbreviations used: Glc*p*, glucopyranose; Ac, acetyl; Rha*p*, rhamnopyranose.

For the further detailed studies, samples of the individual flavonoids previously isolated from *C. officinalis* flowers were used [28,34,35,36]. The acetylcholinesterase inhibition assay was performed using a spectrophotometric method [37]. A number of compounds were investigated including HPLC-detected isorhamnetin and quercetin derivatives, 3-*O*-glucosides, 3-*O*-(6′′-acetyl)-glucosides, 3-*O*-(2′′-rhamnosyl)-glucosides, 3-*O*-(6′′-rhamnosyl)-glucosides, 3-*O*-(2′′,6′′-di-rhamnosyl)- glucosides and 3-*O*-(2′′-rhamnosyl)-rhamnosides. Additionally minor flavonoids of *C. officinalis* flowers were included for analysis such as 3-*O*-(2′′-acetyl)-glucosides, 3-*O*-(2′′,6′′-di-acetyl)-glucosides, 3-*O*-(3′′-rhamnosyl)-glucosides, 3-*O*-(4′′-rhamnosyl)-glucosides and 3-*O*-rhamnosides (Figure 3).

The inhibitory activity of isorhamnetin glycosides expressed as IC_50_ was from 51.26–98.45 μM with maximal potency for 3-*O*-(2′′,6′′-di-acetyl)-glucoside and minimal potency for 3-*O*-(2′′,6′′-di-rhamnosyl)-glucoside; the latter was the dominant compound in the plant object investigated (Table 3). The isorhamnetin inhibition power was highest (24.18 μM), demonstrating the negative influence of 3-*O*-glycosylation on the anti-acetylcholinesterase activity of flavonoids. However, according to the literature data, the hydroxyl group at position C-3 is not involved in the hydrogen bonding with acetylcholinesterase. Hydroxylation at these positions is important for metal chelation, antioxidant effect and the prevention of Aβ aggregation [38,39,40]. Attaching a rhamnosyl moiety to an isorhamnetin skeleton resulted in the formation of a more active compound compared with a glucosyl analogue (IC_50_ isorhamnetin-3-*O*-rhamnoside 73.96 μM vs. IC_50_ isorhamnetin-3-*O*-glucoside 89.04 μM). Substitution of hydroxyl groups in the carbohydrate fragment with acetyls in the 2′′- and/or 6′′-positions increased the activity of the resultant compound. Comparing the activity of isorhamnetin-3-*O*-glucoside and its rhamnosylated analogues demonstrated the reduction of the power of the latter, and the position of the rhamnosyl moiety (2′′, 3′′, 4′′ or 6′′) has a weak influence on the severity of inhibition. Quercetin glycosides were 20–35% more active than the same analogues of isorhamnetin, and the general character of the structure-activity dependence was close.

The high potency of quercetin and its glucosides to inhibit acetylcholinesterase has been described previously in many works [41,42,43]. Information about isorhamnetin derivatives is not so common. However, the results obtained allow us to conclude that isorhamnetin and its glucosides are natural components with anti-acetylcholinesterase potency. Previously, some authors mentioned that all flavonols possess a similar binding pattern in the active site of acetylcholinesterase [40]. The general interactions were found to be between the flavonol skeleton and enzyme active sites. Interaction of the A-ring-involved functional groups was described as between the hydroxyl group at the C-7 position and Asp74 or Tyr72 residues, forming a hydrogen bond [44]. Hydroxylation of the B-ring at C-3′ and C-4′ may also form a hydrogen bond with the residues Ser203 and Gly121 and often with Gly122. The possibility of interaction between the C-4-keto function of C-rings and the residue Phe295 was shown. The structural differences between quercetin and isorhamnetin are only in the methoxy group in the 3′-position in the B-ring of the latter compound. Based on these data, it can be concluded that both substances may decrease the activity of acetylcholinesterase by binding to its active sites.

### 2.4. Flavonoid Content in Marigold Flower Products

In order to evaluate the possible anti-cholinesterase activity of commercial marigold products, we conducted HPLC analysis of flavonoids (quercetin and isorhamnetin derivatives) of 16 marigold tea batches (Table 4). The samples analyzed were purchased from four regions of Russia: Central federal district, five samples (01, 09, 10, 15, 16), Siberian federal district, eight samples (03, 05, 06, 07, 08, 11, 13, 14), Southern federal district, one sample (02), and Ural federal district, one sample (12). Thus, the acquired commercial samples were grown in different regions of the country. In addition, one sample was acquired in the Republic of Uzbekistan (sample 03). The maximal total flavonoid content (19.85 mg/g) was observed in sample 15, and the minimal flavonoid content (7.18 mg/g) was observed in sample 13. The content of isorhamnetin derivatives was 6.8–16.2-times higher than the content of quercetin derivatives.

The data obtained demonstrated the dominance of typhaneoside (isorhamnetin derivative) in all analyzed marigold tea batches, from 3.88 mg/g (sample 13) to 10.45 mg/g (sample 03). In turn, the dominance of both manghaslin, from 0.34 mg/g (sample 16) to 0.72 mg/g (sample 03), and calendoflavobioside, from 0.21 mg/g (sample 13) to 0.96 mg/g (sample 05), was noticed in the analysis of quercetin derivatives. It should be noted that the minor flavonoids quercetin-3-*O*-(2′′-rhamnosyl)-rhamnoside and quercetin-3-*O*-(6′′-acetyl)-glucoside were not detected or revealed in trace amounts in some samples (07, 08, 09, 11, 12, 14, 16). Thus, the commercial sample 15 was chosen for further investigation due to the high content of quercetin and isorhamnetin derivatives.

Given the widespread use of preparations from *C. officinalis* in therapeutic practice, we also investigated the qualitative and quantitative content of phenolic compounds in four medicinal forms, including commercial ethanol-containing forms (liquid extract and tincture) and aqueous forms (decoction and infusion) as frequently applied home-made preparations.

The qualitative composition of the analyzed preparations from *C. officinalis* was similar to those of native plant material (Table 5). This indicates the safety of the componential profile of the analyzed preparations within the manufacturing process. The most enriched liquid formulation was liquid extract, characterized by 3.10 mg/mL of total flavonoid content. Tincture, decoction and infusion are dosage forms prepared by low technology, which affects the composition of the resulting product. The content of flavonoids in tincture, decoction and infusion was significantly lower (0.70, 0.57 and 0.45 mg/mL, respectively) than in liquid extract. In all liquid preparations, a predominance of the quercetin derivative calendoflavobioside and isorhamnetin derivative typhaneoside was observed.

Information on the acceptable intake of various liquid preparations [45] allowed us to calculate values for maximal daily consumption of flavonoids after the application of the mentioned marigold preparations. The results obtained showed that despite the archaic character of aqueous preparations of *C. officinalis*, their application maximized the intake values of flavonoids compared with ethanol formulations. Thus, the intake from daily dosage of marigold decoction (142.50 mg/day) increased flavonoid consumption by 45-times compared to a daily dose of tincture (3.15 mg/day). Despite the high content of flavonoids in liquid extract, daily uptake (9.30 mg/day) is 15.3-times lower than for consumption of a daily dose of decoction.

These data demonstrate the possibility of adequate substitution of liquid extract or tincture by infusions or decoctions when it is not appropriate to administer ethanol-containing formulations (children’s therapy, allergy to ethanol, etc.).

## 3. Materials and Methods

### 3.1. Chemicals

The following chemicals were purchased from Extrasynthese (Lyon, France): 3-*O*-caffeoylquinic acid (chlorogenic acid; Cat. No. 4991, ≥99%); caffeic acid (Cat. No. 6034, ≥99%); 3,5-di-*O*-caffeoylquinic acid (Cat. No. 4946, ≥97%); isorhamnetin (Cat. No. 1120, ≥99%); isorhamnetin-3-*O*-glucoside (Cat. No. 1228, ≥95%); isorhamnetin-3-*O*-rutinoside (narcissin; Cat. No. 1333, ≥99%); quercetin-3-*O*-rutinoside (rutin; cat. No. 1139, ≥99%); quercetin-3-O-rhamnoside (quercitrin; Cat. No. 1236, ≥98.5%); Sigma-Aldrich (St. Louis, MO, USA): acetylcholinesterase from *Electrophorus electricus* (electric eel) (Cat. No. C2888, Type V-S, ≥1000 units/mg protein), Fast Blue B salt (Cat. No. D9805, dye content 95%), lithium perchlorate (Cat. No. 431567, ≥99.99%), α-naphthyl acetate (Cat. No. N8505, ≥98%), neostigmine bromide (Cat. No. 2001, ≥98%), perchloric acid (Cat. No. 311421, ≥70%, 99.999% trace metals basis), quercetin (Cat. No. Q0125, ≥98%), quercetin-3-*O*-glucoside (Cat. No. 16654, ≥98%), sodium dodecyl sulfate (Cat. No. 436143, ≥99%). 1,5-Di-*O*-caffeoylquinic acid, 4,5-di-*O*-caffeoylquinic acid, isorhamnetin-3-*O*-(2′′-acetyl)-glucoside, isorhamnetin-3-*O*-(6′′-acetyl)-glucoside, isorhamnetin-3-*O*-(2′′,6′′-di-acetyl)-glucoside, isorhamnetin- 3-*O*-(2′′-rhamnosyl)-glucoside (calendoflavoside), isorhamnetin-3-*O*-(3′′-rhamnosyl)-glucoside, isorhamnetin-3-*O*-(4′′-rhamnosyl)-glucoside, isorhamnetin-3-*O*-(2′′,6′′-di-rhamnosyl)-glucoside (typhaneoside), isorhamnetin-3-*O*-rhamnoside, isorhamnetin-3-*O*-(2′′-rhamnosyl)-rhamnoside (calendoflaside), quercetin-3-*O*-(2′′-acetyl)-glucoside, quercetin-3-*O*-(6′′-acetyl)-glucoside, quercetin-3-*O*-(2′′,6′′-di-acetyl)-glucoside, quercetin-3-*O*-(2′′-rhamnosyl)-glucoside (calendoflavobioside), quercetin-3-*O*-(3′′-rhamnosyl)-glucoside, quercetin-3-*O*-(4′′-rhamnosyl)- glucoside, 3-*O*-(2′′,6′′-di-rhamnosyl)-glucoside (manghaslin) and quercetin-3-*O*-(2′′-rhamnosyl)- rhamnoside were isolated previously from *C. officinalis* [28,34,35,36].

### 3.2. Plant Material

Plants of *Calendula officinalis* L. in twenty three double-flowered varieties (Amber Bay, Big Orange, Cardinal, Egypt Sun, Fiesta, Flame Dancer, Gavrish, Geisha Girl, Gitana Orange, Golden Imperator, Golden Prince, Green Heart Orange, Honey Cardinal, Indian Prince, Jiga-Jiga, Lemon Juice, Orange Balls, Orange King, Radio, Red Black Centered, Rose Surprise, Touch of Red, Tutti-Frutti) were grown from authenticated seeds obtained from Tsitsin’s Main Botanical Garden of the Russian Academy of Science (Moscow, Russian) by cultivation in the fields of the Botanical Garden of the North-Eastern Federal University (NEFU, Yakutsk, Russian). The flowers were collected in the middle of August 2016 and then dried in vacuo at 40 °C (12  h) and stored at 4 °C in the Institute of General and Experimental Biology Plant Repository. Commercial samples of marigold tea were purchased from Pharmaceutical Company Magnolia (Moscow, Russia; Batch No. 080616; Sample 01), Company Phytopharm (Anapa, Russia; Batch No. 03116; Sample 02), Shalfey Ltd. (Irkutsk, Russia; Batch No. 010116; Sample 03), Zamona Rano Ltd. (Nomdanak, Uzbekistan; Batch No. 050616; Sample 04), Eastern Medicine Ltd. (Ulan-Ude, Russia; Batch No. 150816; Sample 05), Company Khorst Ltd. (Barnaul, Russia; Batch No. 060216; Sample 06), Altaivitaminy Ltd. (Biisk, Russia; Batch No. 1931116; Sample 07), Company Tayga Produkt (Angarsk, Russia; Batch No. 010716; Sample 08), Company Ivan-Chai (Moscow, Russia; Batch No. 021116; Sample 09), LeksPlus Ltd. (Khimki, Russia; Batch No. 01016; Sample 10), TsSI Ltd. (Barnaul, Russia; Batch No. 100116; Sample 11), Pharmaceutical Company Zdorov’e (Magnitogorsk, Russia; Batch No. 040416; Sample 12), Ortilia Ltd. (Irkutsk, Russia; Batch No. 01066; Sample 13), Company Travy Daurii (Chita, Russia; Batch No. 250616; Sample 14), Public Corporation Krasnogorskleksredstva (Moscow, Russia; Batch No. 150616; Sample 15), ST-Medipharm (Moscow, Russia; Batch No. 010516; Sample 16). Commercial samples of *C. officinalis* preparations were purchased in Company Flora Kavkaza (tincture, Pregradnaya, Russia; Batch No. 090816) and Company Arura (liquid extract, Ulan-Ude, Russia; Batch No. 100916).

### 3.3. Sample Preparation for the Extraction of Total Phytochemicals and Anti-acetylcolinesterase Acitivity Determination

For preparation of total extracts of twenty three varieties of *C. officinalis* with maximal content of basic groups of compounds (essential oils, carotenoids, triterpenoids, flavonoids, phenylpropanoids and polysaccharides), plant material was extracted by following solvents, as 96% ethanol for extraction of essential oils, carotenoids and triterpenoids; 60% ethanol as an optimal solvent for flavonoids and phenylpropanoids; water as an optimal solvent for polysaccharides. Accurately-weighed *C. officinalis* plant sample (100 g) was placed in a conical flask. Then, 1500 mL of the 96% ethanol solution were added, and the mixture was extracted twice in an ultrasonic bath for 90 min at 45 °C. The extracted solution A was filtered through a cellulose filter. The plant residue was repeatedly extracted by 60 % ethanol and water in the same conditions receiving extracts B and C, accordingly. Finally extracts A, B and C were combined and evaporated in vacuo until dryness using a rotary evaporator. The total extracts were stored at 4 °C until further chemical composition analysis and anti-acetylcholinesterase activity microplate assay.

### 3.4. Chemical Composition Analytical Methods

The essential oil content was determined gravimetrically after hydrodistillation in a Clevenger-type apparatus for 150 min [46]. The concentration of carotenoids was estimated as β-carotene equivalent using the spectrophotometric method at 450 nm in preliminary saponified extracts [47]. The total triterpenoid content was determined by HPTLC-densitometric analysis after acidic hydrolysis in 7% HCl/acetone media as oleanolic acid equivalents [48]. The flavonoid content was estimated as narcissin equivalents after spectrophotometric procedure after 5% AlCl_3_ addiction [49]. The phenylpropanoid content was determined by the colorimetric Arnow method using 3-*O*-caffeoylquinic acid as the standard [50]. The polysaccharides content was determined by the spectrophotometric anthrone-sulfuric acid method with galactose as the standard [51].

### 3.5. Anti-Acetylcholinesterase Activity Microplate Assay

The acetylcholinesterase inhibition assay was performed using a spectrophotometric microplate assay [37]. The reaction mixture consisted of 235.7 µL of 0.1 M phosphate buffer pH 7.4, 20 µL of a solution of acetylcholinesterase (final concentration 0.1 U/mL in 0.1 M phosphate buffer pH 7.4), 2.7 µL of test compound or plant extract solutions in dimethyl sulfoxide (DMSO). The controls contained the corresponding volume of DMSO of test compound solutions. The enzymatic reaction was initiated by the addition of 1.6 µL of α-naphthyl acetate solution in DMSO. After mixing for 90 s and incubation at 25 °C for 90 s, the reaction was stopped with 20 µL of a 5% solution of sodium dodecyl sulfate (SDS) in water. The color was developed with 20 µL of Fast Blue B salt solution (final concentration 0.17 mM in water). The method was realized in 96-well plates. The microplate was read by a Bio-Rad microplate reader Model 3550 UV. Enzyme activity and inhibition were quantified by determination of the absorbance at 600 nm after the formation of the purple-colored diazonium dye as a percentage. A control sample was considered to have 100% was carried out using the same volume of DMSO instead of tested compound (or plant extract). The percentage of inhibition was calculated relative to a control sample, for which the anti-acetylcholinesterase activity was assessed under identical conditions, but in the absence of the test compound, using the expression:

% Inhibition = {[(*A*_CE_ − *A*_C_) − (*A*_TE_ − *A*_T_)]/(*A*_CE_ − *A*_C_)} × 100%

where *A*_CE_ is the absorbance at 600 nm of the control sample with enzyme; *A*_C_ is the absorbance at 600 nm of the control sample without enzyme; *A*_TE_ is the absorbance at 600 nm of the test compound (or plant extract) with enzyme; *A*_T_ is the absorbance at 600 nm of the test compound (or plant extract) without enzyme. Linear equation indicating the correlation between the common logarithm of the compound concentration (μM) and percentage of acetylcholinesterase inhibition (%) was build, and from which the IC_50_ values (concentration that inhibits 50% of acetylcholinesterase activity) of tested compounds (or plant extracts) were extrapolated.

### 3.6. Microcolumn Reversed-Phase High-Performance Liquid Chromatography with Ultraviolet Detection (MC-RP-HPLC-UV) Conditions

MC-RP-HPLC-UV experiments were performed on an Econova MiLiChrom A-02 microcolumn chromatograph (Novosibirsk, Novosibirsk Oblast, Russia) coupled with UV-detector, using a ProntoSIL-120-5-C18 AQ column (1 × 75 mm, Φ 1 μm; Metrohm AG, Herisau, Switzerland); the column temperature was 35 °C. Eluent A was 0.2 M LiClO_4_ in 0.006 M HClO_4_, and Eluent B was acetonitrile. The injection volume was 1 μL, and elution was at 100 μL/min. Gradient program: 0–7.5 min 11–18% B, 7.5–13.5 min 18% B, 13.5–15 min 18–20% B, 15–18 min 20–25% B, 18–24.0 min 25% B, 24–30.0 min 25–100% B. UV-Spectral data from all peaks were accumulated in the range of 200–600 nm, and chromatograms were recorded at 330 nm.

MC-HPLC-UV quantification experiments were carried out at the same chromatographic conditions with UV-detection at 350 nm. Stock solutions of standards were made by accurately weighing 1 mg samples of 3-*O*-caffeoylquinic acid (chlorogenic acid), caffeic acid, 1,5-di-*O*-caffeoylquinic acid, 4,5-di-*O*-caffeoylquinic acid, 3,5-di-*O*-caffeoylquinic acid, isorhamnetin, isorhamnetin-3-*O*-glucoside, isorhamnetin-3-*O*-rutinoside (narcissin), isorhamnetin-3-*O*-(2′′-acetyl)-glucoside, isorhamnetin-3-*O*-(6′′-acetyl)-glucoside, isorhamnetin-3-*O*-(2′′,6′′-di-acetyl)-glucoside, isorhamnetin-3-*O*-(2′′-rhamnosyl)-glucoside (calendoflavoside), isorhamnetin-3-*O*-(3′′-rhamnosyl)-glucoside, isorhamnetin-3-*O*-(4′′-rhamnosyl)-glucoside, isorhamnetin-3-*O*-(2′′,6′′-di-rhamnosyl)-glucoside (typhaneoside), isorhamnetin-3-*O*-rhamnoside, isorhamnetin-3-*O*-(2′′-rhamnosyl)-rhamnoside (calendoflaside), quercetin, quercetin-3-*O*-rutinoside (rutin), quercetin-3-*O*-rhamnoside (quercitrin), quercetin-3-*O*-glucoside, quercetin-3-*O*-(2′′-acetyl)-glucoside, quercetin-3-*O*-(6′′-acetyl)-glucoside, quercetin-3-*O*-(2′′,6′′-di-acetyl)-glucoside, quercetin-3-*O*-(2′′-rhamnosyl)-glucoside (calendoflavobioside), quercetin-3-*O*-(3′′-rhamnosyl)-glucoside, quercetin-3-*O*-(4′′-rhamnosyl)-glucoside, 3-*O*-(2′′,6′′-di-rhamnosyl)-glucoside (manghaslin), quercetin-3-*O*-(2′′-rhamnosyl)-rhamnoside and separately dissolving them in 1 mL of 20 % DMSO solution in methanol in a volumetric flask. The appropriate amounts of stock solutions were diluted with methanol in order to obtain standard solutions containing 0.25–1.00 mg/mL. As all of the compounds used for quantification were well separated under the experimental conditions, mixtures of standards were analyzed. Prepared solutions were stored at 4 °C for no more than 72 h. The results are presented as the mean values ± SD (standard deviations) of three replicates.

For preparation of 60% ethanol extract, used for MC-RP-HPLC-UV and MC-RP-HPLC-UV-ESI-MS analysis, accurately-weighed *C. officinalis* plant sample of Greenheart Orange variety (100 g) was placed in a conical flask. Then, 1500 mL of the 60% ethanol solution were added, and the mixture was extracted twice in an ultrasonic bath for 90 min at 45 °C. The extracted solutions were filtered through a cellulose filter and evaporated in vacuo until dryness using a rotary evaporator. For the preparation of 60% ethanol extract solution, an accurately weighed dry extract of *C. officinalis* (10 mg) was placed in an Eppendorf tube; 1 mL of 60% ethanol was added; and the mixture was weighed again. Then, the sample was extracted in an ultrasonic bath for 10 min at 40 °C. After cooling, the resultant extract was filtered through a 0.22-μm polytetrafluoroethylene (PTFE) syringe filter before injection into the HPLC system for analysis.

For the preparation of the sample solution of commercial of marigold tea batches (samples 01–16), accurately-weighed plant sample (1 g) was placed in conical flasks. Then, 100 mL of the 60% ethanol solution were added, and the mixture was extracted in an ultrasonic bath for 30 min at 45 °C. The extracted solution was filtered through a 0.22-μm PTFE syringe filter before injection into the HPLC system for analysis.

For the preparation of the decoction, accurately-weighed *C. officinalis* plant Sample 15 (1 g) was placed in conical flasks. Then, 100 mL of distilled water was added, and the sample was heated on a hotplate and boiled for 10 min. The mixture was left to stand at room temperature for 15 min, then filtered under reduced pressure and made up to 100 mL in a volumetric flask. The resultant decoction was filtered through a 0.22-μm PTFE syringe filter before injection into the HPLC system for analysis.

For the preparation of infusion, accurately-weighed *C. officinalis* plant Sample 15 (1 g) was placed in conical flasks. Then, 100 mL of boiled distilled water was added. The sample was then stirred for 40 min. Then, the mixture was filtered under reduced pressure and made up to 100 mL in a volumetric flask. The resultant infusion was filtered through a 0.22-μm PTFE syringe filter before injection into the HPLC system for analysis.

### 3.7. Microcolumn Reversed-Phase High-Performance Liquid Chromatography with Electrospray Ionization Mass Spectrometry Detection (MC-RP-HPLC-ESI-MS) Conditions

MC-RP-HPLC-ESI-MS experiments were performed on an Econova MiLiChrom A-02 microcolumn chromatograph (Novosibirsk, Novosibirsk Oblast, Russia) coupled with triple-quadrupole electrospray ionization mass-spectrometer LCMS 8050 (Shimadzu, Columbia, MD, USA). LC conditions were the same as described in Section 3.5. MS conditions were: ionization mode, ESI (negative for phenylpropanoids and positive for flavonoids); electrospray ionization (ESI) interface temperature, 300 °C; desolvation line temperature, 250 °C; heat block temperature, 400 °C; nebulizing gas flow (N_2_), 3 L/min; heating gas flow (air), 10 L/min; heating gas flow (N_2_), 10 L/min; collision-induced dissociation gas (Ar) pressure, 270 kPa; Ar flow, 0.3 mL/min; capillary voltage, 3 kV. The full scan mass covered the range from *m*/*z* 100 up to *m*/*z* 1900.

### 3.8. HPLC Activity-Based Profiling

HPLC activity-based profiling was realized using the post-chromatographic reaction of HPLC-eluates with the acetylcholinesterase/α-naphthyl acetate/Fast Blue B salt model system. Aliquots (100 µL) of extract solution (10 mg/mL) were separated under analytical HPLC conditions (Section 2.4). The eluates (50 µL) were collected every 30 s using an automated fraction collector (Econova) in 96-well plates, then dried under a N_2_-steam and redissolved in 2.7 µL of DMSO. To each sample, 235.7 µL of 0.1 M phosphate buffer pH 7.4 and 20 µL of a solution acetylcholinesterase (final concentration 0.1 U/mL in 0.1 M phosphate buffer) and 1.6 µL of α-naphthyl acetate solution in DMSO were added. After mixing for 90 s and incubation at 25 °C for 90 s, 20 µL of a 5% solution of sodium dodecyl sulfate (SDS) in water and 20 µL of Fast Blue B salt solution in water (final concentration 0.17 mM) were added. The microplate was read by a Bio-Rad microplate reader Model 3550 UV (Bio-Rad Labs, Richmond, CA, USA). Enzyme activity and inhibition were quantified by determination of the absorbance at 600 nm after the formation of the purple-colored diazonium dye as a percentage. The percentage of inhibition was calculated relative to a control sample, for which the anti-acetylcholinesterase activity was assessed under identical conditions, but in the absence of the test compound.

### 3.9. Statistical Analysis

All experiments were done in triplicates, and the results were given as the mean ± standard deviation (SD). One-way analysis of variance (ANOVA) with a post hoc least significant difference test was used to determine significance (*p* ≤ 0.05) with Statistica 5.5 software (Dell Technologies Inc., Round Rock, TX, USA) together with the correlation matrix with the use of elementary statistics.

## 4. Conclusions

In this study, we investigated the anti-acetylcholinesterase potential of *C. officinalis* flowers introduced into Siberia based on their chemical composition, which has not been reported previously. The effect of total flavonoid content on the anti-acetylcholinesterase activity of ethanolic extracts was significant. HPLC activity-based profiling data suggested that the quercetin and isorhamnetin derivatives possess strong and moderate anti-acetylcholinesterase activity, respectively. Structural characteristics that may contribute to the understanding of the bioactivity of quercetin and isorhamnetin glycosides were identified. The presence of a rhamnosyl moiety and acetyl groups at the 2′′- and/or 6′′-position of the carbohydrate function of a flavonoid skeleton has an influence on their anti-acetylcholinesterase properties. In addition, the flavonoid content of 16 commercial batches of marigold flowers and four liquid preparations was analyzed. The levels of isorhamnetin and quercetin derivatives detected in samples of marigold tea were 6.57–17.86 and 0.61–2.08 mg/g, respectively, and in liquid preparations were 0.37–2.62 and 0.08–0.48 mg/mL, respectively. On the basis of this study, it can be concluded that quercetin and isorhamnetin glycosides from *C. officinalis* flowers have the potential to be promising candidates for the development of anti-acetylcholinesterase agents.

## Figures and Tables

**Table 1:** Chemical composition and anti-acetylcholinesterase activity (AChA) of total extracts of 23 varieties of *C. officinalis* flowers (mg/g dry weight (DW) ± standard deviation (SD)) ^1^.

Variety	Essential Oil	Carotenoids	Triterpenes	Flavonoids	Phenylpropanoids	Polysaccharides	AChA, IC_50_, μg/mL
Amber Bay	1.12 ± 0.04	4.12 ± 0.10	25.17 ± 0.48	18.53 ± 0.42	19.45 ± 0.33	30.27 ± 0.48	133.9 ± 4.3
Big Orange	2.65 ± 0.12	6.56 ± 0.17	39.11 ± 0.82	26.79 ± 0.56	18.54 ± 0.35	35.62 ± 0.64	123.7 ± 3.8
Cardinal	0.32 ± 0.01	8.29 ± 0.19	27.63 ± 0.50	12.65 ± 0.29	25.09 ± 0.40	25.11 ± 0.50	197.2 ± 6.5
Egypt Sun	1.82 ± 0.09	5.47 ± 0.12	65.70 ± 1.31	19.24 ± 0.38	7.30 ± 0.14	39.16 ± 0.67	123.8 ± 4.2
Fiesta	1.43 ± 0.05	10.35 ± 0.27	37.16 ± 0.71	16.35 ± 0.38	15.17 ± 0.27	42.54 ± 0.68	152.4 ± 5.2
Flame Dancer	2.93 ± 0.12	7.59 ± 0.20	10.28 ± 0.19	10.52 ± 0.24	22.63 ± 0.41	11.97 ± 0.22	187.2 ± 6.2
Gavrish	2.20 ± 0.10	5.22 ± 0.12	12.04 ± 0.24	29.37 ± 0.67	20.01 ± 0.36	28.14 ± 0.59	104.2 ± 3.2
Geisha Girl	2.55 ± 0.12	6.97 ± 0.16	20.33 ± 0.43	20.11 ± 0.44	24.16 ± 0.56	22.67 ± 0.43	137.3 ± 4.5
Gitana Orange	0.93 ± 0.04	6.31 ± 0.15	20.16 ± 0.44	32.54 ± 0.75	4.93 ± 0.09	19.27 ± 0.39	125.2 ± 4.3
Golden Imperator	1.12 ± 0.05	4.09 ± 0.11	42.82 ± 0.86	26.68 ± 0.62	33.47 ± 0.60	17.06 ± 0.34	153.9 ± 5.5
Golden Prince	2.43 ± 0.12	3.31 ± 0.09	14.15 ± 0.25	25.14 ± 0.58	6.07 ± 0.12	23.69 ± 0.50	161.9 ± 5.5
Green Heart Orange	0.89 ± 0.04	8.61 ± 0.22	15.80 ± 0.25	46.87 ± 1.03	30.24 ± 0.51	39.02 ± 0.70	63.5 ± 2.1
Honey Cardinal	1.03 ± 0.04	7.22 ± 0.15	30.70 ± 0.52	37.18 ± 0.78	26.12 ± 0.52	44.15 ± 0.79	105.2 ± 3.6
Indian Prince	1.47 ± 0.07	5.14 ± 0.11	37.93 ± 0.72	17.25 ± 0.36	18.69 ± 0.39	25.10 ± 0.40	169.5 ± 6.1
Jiga-Jiga	3.04 ± 0.15	5.22 ± 0.15	16.72 ± 0.27	11.67 ± 0.23	8.53 ± 0.14	27.82 ± 0.45	223.9 ± 7.6
Lemon Juice	2.47 ± 0.10	5.67 ± 0.14	53.74 ± 1.07	21.38 ± 0.49	25.84 ± 0.52	19.82 ± 0.36	150.1 ± 5.4
Orange Balls	1.45 ± 0.06	7.14 ± 0.17	25.37 ± 0.51	19.53 ± 0.43	11.47 ± 0.21	16.37 ± 0.31	179.5 ± 5.7
Orange King	1.39 ± 0.05	8.26 ± 0.22	53.41 ± 1.07	16.38 ± 0.33	16.72 ± 0.35	38.25 ± 0.77	202.4 ± 7.3
Radio	1.81 ± 0.09	5.98 ± 0.14	61.37 ± 1.29	18.42 ± 0.40	10.83 ± 0.22	22.16 ± 0.42	133.6 ± 4.5
Red Black Centered	2.12 ± 0.11	6.77 ± 0.15	11.39 ± 0.23	16.34 ± 0.32	8.42 ± 0.17	20.38 ± 0.37	175.3 ± 6.3
Rose Surprise	0.73 ± 0.03	11.39 ± 0.29	33.44 ± 0.54	19.63 ± 0.39	29.06 ± 0.64	11.09 ± 0.22	122.2 ± 4.5
Touch of Red	1.64 ± 0.07	2.63 ± 0.06	35.25 ± 0.60	28.16 ± 0.54	28.24 ± 0.48	15.23 ± 0.30	105.6 ± 3.3
Tutti-Frutti	1.73 ± 0.08	9.23 ± 0.23	28.16 ± 0.45	18.37 ± 0.42	18.28 ± 0.29	18.09 ± 0.36	169.8 ± 5.8

^1^ Averages ± standard deviation were obtained from five different experiments. Reference compounds: neostigmine (positive control; active, IC_50_ = 0.75 ± 0.01 μg/mL), NaCl (negative control; inactive).

**Table 2:** HPLC parameters, UV and electrospray ionization mass spectrometry (ESI-MS) data and the content of phenylpropanoids, quercetin and isorhamnetin derivatives in 60% ethanol extract of *C. officinalis* (Greenheart Orange variety).

Compound	*t*_R_, min	UV, λ_max_, nm	ESI-MS, *m*/*z*	Content, mg/g DW ± SD ^1^
Phenylpropanoids				
3-*O*-Caffeoylquinic acid	6.83	324	353 [M − H]^−^, 191, 179, 135	3.32 ± 0.08
Caffeic acid	7.90	325	179 [M − H]^−^, 135	0.92 ± 0.02
3,5-Di-*O*-caffeoylquinic acid	15.31	333	515 [M − H]^−^, 353, 191, 179, 135	1.16 ± 0.03
1,5-Di-*O*-caffeoylquinic acid	17.52	332	515 [M − H]^−^, 353, 191, 179	3.03 ± 0.07
4,5-Di-*O*-caffeoylquinic acid	20.37	331	515 [M − H]^−^, 353, 179	1.02 ± 0.02
Flavonoids. Quercetin derivatives				
Manghaslin	10.63	255, 356	757 [M + H]^+^, 611, 465, 303	12.62 ± 0.32
Calendoflavobioside	12.06	255, 356	611 [M + H]^+^, 465, 303	10.12 ± 0.25
Rutin	13.18	255, 356	611 [M + H]^+^, 465, 303	2.26 ± 0.05
Isoquercitrin	13.87	257, 356	465 [M + H]^+^, 303	0.66 ± 0.01
Quercetin-3-*O*-(2″-ramnosyl)-rhamnoside	14.16	259, 353	595 [M + H]^+^, 449, 303	0.49 ± 0.01
Quercetin-3-*O*-(6″-acetyl)-glucoside	16.14	261, 352	507 [M + H]^+^, 303	0.60 ± 0.01
Flavonoids. Isorhamnetin derivatives				
Typhaneoside	10.51	254, 356	771 [M + H]^+^, 625, 479, 317	42.46 ± 1.10
Calendoflavoside	14.63	255, 357	625 [M + H]^+^, 479, 317	6.43 ± 0.16
Narcissin	18.46	255, 355	625 [M + H]^+^, 479, 317	12.92 ± 0.33
Isorhamnetin-3-*O*-glucoside	19.09	258, 361	479 [M + H]^+^, 317	1.79 ± 0.04
Calendoflaside	19.82	253, 354	609 [M + H]^+^, 463, 317	0.33 ± 0.01
Isorhamnetin-3-*O*-(6″-acetyl)-glucoside	22.21	253, 354	521 [M + H]^+^, 317	1.86 ± 0.04
			Total content:	
			phenylpropanoids	9.45
			quercetin derivatives	26.75
			isorhamnetin derivatives	65.79
			flavonoids	92.54

^1^ Averages ± standard deviation were obtained from three different experiments.

**Table 3:** Anti-acetylcholinesterase activity of isorhamnetin, quercetin and its glycosides (IC_50_, μM ± SD) ^1^.

Carbohydrate Unit	Isorhamnetin	Quercetin
-	24.18 ± 0.74	14.37 ± 0.34
3-*O*-Glc*p*	89.04 ± 2.49	70.12 ± 1.82
3-*O*-(2″-Ac)-Glc*p*	70.85 ± 1.84	48.01 ± 1.20
3-*O*-(6″-Ac)-Glc*p*	68.22 ± 1.71	45.16 ± 1.12
3-*O*-(2″,6″-di-Ac)-Glc*p*	51.26 ± 1.53	36.47 ± 1.02
3-*O*-(2″-Rha*p*)-Glc*p*	94.27 ± 2.82	71.86 ± 1.94
3-*O*-(3″-Rha*p*)-Glc*p*	91.16 ± 2.73	69.15 ± 1.84
3-*O*-(4″-Rha*p*)-Glc*p*	92.07 ± 2.85	70.35 ± 1.90
3-*O*-(6″-Rha*p*)-Glc*p*	97.32 ± 2.91	72.09 ± 2.04
3-*O*-(2″,6″-di-Rha*p*)-Glc*p*	98.45 ± 3.04	94.92 ± 2.65
3-*O*-Rha*p*	73.96 ± 2.14	48.80 ± 1.26
3-*O*-(2″-Rha*p*)-Rha*p*	84.90 ± 2.37	67.91 ± 1.76

^1^ Averages ± standard deviation were obtained from three different experiments. Reference compounds: neostigmine (positive control; active, IC_50_ = 3.37 ± 0.09 μM), NaCl (negative control; inactive). Abbreviations used: Glc*p*, glucopyranose; Ac, acetyl; Rha*p*, rhamnopyranose.

**Table 4:** Content of quercetin and isorhamnetin derivatives in 16 marigold tea batches (01–16; mg/g DW ± SD) ^1^.

Compound	01	02	03	04	05	06	07	08
Quercetin derivatives								
Manghaslin	0.21 ± 0.00	0.43 ± 0.01	0.72 ± 0.02	0.27 ± 0.00	0.52 ± 0.01	0.28 ± 0.00	0.30 ± 0.01	0.33 ± 0.00
Calendoflavobioside	0.54 ± 0.01	0.71 ± 0.02	0.38 ± 0.01	0.35 ± 0.01	0.96 ± 0.02	0.32 ± 0.01	0.40 ± 0.01	0.38 ± 0.00
Rutin	0.18 ± 0.00	0.23 ± 0.00	0.18 ± 0.00	0.27 ± 0.00	0.25 ± 0.00	0.06 ± 0.00	0.08 ± 0.00	0.11 ± 0.00
Isoquercitrin	0.15 ± 0.00	0.14 ± 0.00	0.05 ± 0.00	0.07 ± 0.00	0.19 ± 0.00	0.02 ± 0.00	0.02 ± 0.00	0.06 ± 0.00
Quercetin-3-*O*-(2″-Rha)-Rha	0.03 ± 0.00	0.03 ± 0.00	0.04 ± 0.00	0.01 ± 0.00	0.05 ± 0.00	0.03 ± 0.00	0.00 ± 0.00	0.00 ± 0.00
Quercetin-3-*O*-(6″-Ac)-Glc	0.16 ± 0.00	0.09 ± 0.00	0.04 ± 0.00	0.17 ± 0.00	0.11 ± 0.00	0.00 ± 0.00	0.04 ± 0.00	0.09 ± 0.00
Subtotal								
Isorhamnetin derivatives								
Typhaneoside	5.01 ± 0.11	5.83 ± 0.14	10.45 ± 0.24	4.17 ± 0.09	7.18 ± 0.16	4.04 ± 0.09	3.94 ± 0.09	6.03 ± 0.14
Calendoflavoside	0.90 ± 0.02	1.11 ± 0.02	1.21 ± 0.03	0.58 ± 0.01	2.14 ± 0.05	0.87 ± 0.02	0.97 ± 0.02	1.34 ± 0.03
Narcissin	3.49 ± 0.08	3.50 ± 0.08	4.19 ± 0.09	4.16 ± 0.09	4.29 ± 0.09	1.85 ± 0.04	1.70 ± 0.04	3.18 ± 0.07
Isorhamnetin-3-*O*-Glc	0.30 ± 0.00	0.35 ± 0.01	0.08 ± 0.00	0.11 ± 0.00	0.29 ± 0.00	0.18 ± 0.00	0.17 ± 0.00	0.15 ± 0.00
Calendoflaside	0.15 ± 0.00	0.15 ± 0.00	0.05 ± 0.00	0.04 ± 0.00	0.14 ± 0.00	0.02 ± 0.00	0.15 ± 0.00	0.04 ± 0.00
Isorhamnetin-3-*O*-(6″-Ac)-Glc	0.68 ± 0.01	0.30 ± 0.01	0.35 ± 0.01	0.57 ± 0.01	0.31 ± 0.01	0.12 ± 0.00	0.14 ± 0.00	0.40 ± 0.01
Subtotal	10.53	11.24	16.33	9.63	14.35	7.08	7.07	11.14
Total flavonoids	11.80	12.87	17.74	10.77	16.43	7.79	7.91	12.11
**Compound**	**09**	**10**	**11**	**12**	**13**	**14**	**15**	**16**
Quercetin derivatives								
Manghaslin	0.37 ± 0.01	0.56 ± 0.02	0.41 ± 0.01	0.39 ± 0.01	0.15 ± 0.00	0.42 ± 0.01	0.49 ± 0.01	0.34 ± 0.01
Calendoflavobioside	0.45 ± 0.01	0.51 ± 0.01	0.47 ± 0.01	0.47 ± 0.01	0.21 ± 0.00	0.24 ± 0.00	0.57 ± 0.01	0.33 ± 0.01
Rutin	0.09 ± 0.00	0.12 ± 0.00	0.11 ± 0.00	0.11 ± 0.00	0.09 ± 0.00	0.28 ± 0.00	0.79 ± 0.02	0.15 ± 0.00
Isoquercitrin	0.07 ± 0.00	0.09 ± 0.00	0.07 ± 0.00	0.06 ± 0.00	0.05 ± 0.00	0.03 ± 0.00	0.07 ± 0.00	0.07 ± 0.00
Quercetin-3-*O*-(2″-Rha)-Rha	0.00 ± 0.00	0.02 ± 0.00	0.00 ± 0.00	0.00 ± 0.00	0.02 ± 0.00	0.00 ± 0.00	0.03 ± 0.00	0.00 ± 0.00
Quercetin-3-*O*-(6″-Ac)-Glc	0.10 ± 0.00	0.11 ± 0.00	0.09 ± 0.00	0.08 ± 0.00	0.09 ± 0.00	0.11 ± 0.00	0.04 ± 0.00	0.04 ± 0.00
Subtotal	1.08	1.41	1.15	1.11	0.61	1.08	1.99	0.93
Isorhamnetin derivatives								
Typhaneoside	5.23 ± 0.12	5.52 ± 0.12	6.24 ± 0.14	6.17 ± 0.14	3.88 ± 0.08	7.89 ± 0.18	8.89 ± 0.20	9.16 ± 0.21
Calendoflavoside	1.35 ± 0.03	1.16 ± 0.03	1.17 ± 0.02	1.20 ± 0.02	0.28 ± 0.00	0.35 ± 0.01	0.36 ± 0.01	0.29 ± 0.00
Narcissin	2.25 ± 0.05	2.42 ± 0.06	2.70 ± 0.06	2.90 ± 0.07	2.00 ± 0.05	4.69 ± 0.11	7.98 ± 0.18	4.82 ± 0.11
Isorhamnetin-3-*O*-Glc	0.14 ± 0.00	0.15 ± 0.00	0.18 ± 0.00	0.20 ± 0.00	0.15 ± 0.00	0.34 ± 0.01	0.11 ± 0.00	0.11 ± 0.00
Calendoflaside	0.15 ± 0.00	0.19 ± 0.00	0.12 ± 0.00	0.17 ± 0.00	0.12 ± 0.00	0.12 ± 0.00	0.09 ± 0.00	0.14 ± 0.00
Isorhamnetin-3-*O*-(6″-Ac)-Glc	0.33 ± 0.01	0.34 ± 0.00	0.35 ± 0.01	0.35 ± 0.01	0.14 ± 0.00	0.39 ± 0.01	0.43 ± 0.01	0.56 ± 0.02
Subtotal	9.45	9.78	10.76	10.99	6.57	13.78	17.86	15.08
Total flavonoids	10.53	11.19	11.91	12.10	7.18	14.86	19.85	16.01

^1^ Averages ± standard deviation were obtained from three different experiments. Abbreviations used: Glc*p*, glucopyranose; Ac, acetyl; Rha*p*, rhamnopyranose.

**Table 5:** Content of quercetin and isorhamnetin derivatives in liquid preparations of *C. officinalis* (mg/mL DW ± SD) ^1^ and daily intake of flavonoids (mg/day).

Compound	Infusion	Decoction	Tincture	Liquid Extract
Quercetin derivatives				
Manghaslin	0.02 ± 0.00	0.02 ± 0.00	0.03 ± 0.00	0.14 ± 0.00
Calendoflavobioside	0.02 ± 0.00	0.03 ± 0.00	0.04 ± 0.00	0.21 ± 0.00
Rutin	0.01 ± 0.00	0.01 ± 0.00	0.02 ± 0.00	0.07 ± 0.00
Isoquercitrin	0.01 ± 0.00	0.01 ± 0.00	0.01 ± 0.00	0.01 ± 0.00
Quercetin-3-*O*-(2″-Rha)-Rha	0.01 ± 0.00	0.01 ± 0.00	0.01 ± 0.00	0.02 ± 0.00
Quercetin-3-*O*-(6″-Ac)- Glc	0.01 ± 0.00	0.01 ± 0.00	0.01 ± 0.00	0.03 ± 0.00
Subtotal	0.08	0.09	0.12	0.48
Isorhamnetin derivatives				
Typhaneoside	0.19 ± 0.01	0.23 ± 0.01	0.26 ± 0.01	1.24 ± 0.03
Calendoflavoside	0.01 ± 0.00	0.01 ± 0.00	0.01 ± 0.00	0.10 ± 0.00
Narcissin	0.14 ± 0.01	0.21 ± 0.01	0.26 ± 0.01	1.09 ± 0.03
Isorhamnetin-3-*O*-Glc	0.01 ± 0.00	0.01 ± 0.00	0.03 ± 0.00	0.15 ± 0.00
Calendoflaside	0.01 ± 0.00	0.01 ± 0.00	0.01 ± 0.00	0.02 ± 0.00
Isorhamnetin-3-*O*-(6″-Ac)-Glc	0.01 ± 0.00	0.01 ± 0.00	0.01 ± 0.00	0.02 ± 0.00
Subtotal	0.37	0.48	0.58	2.62
Total flavonoids	0.45	0.57	0.70	3.10
Daily intake of flavonoids	112.50 ^2^	142.50 ^2^	3.15 ^3^	9.30 ^4^

^1^ Averages ± standard deviation were obtained from three different experiments. Abbreviations used: Glc*p*, glucopyranose; Ac, acetyl; Rha*p*, rhamnopyranose. ^2^ Recommended maximal daily intake of infusions and decoctions, 250 mL. ^3^ Recommended maximal daily intake of tincture, 4.5 mL. ^4^ Recommended maximal daily intake of liquid extract, 3.0 mL.

## Supplementary Materials

Supplementary materials can be found at www.mdpi.com/1422-0067/18/8/1685/s1.
