# Supplementary material for: Isorhamnetin and Quercetin Derivatives as Anti-Acetylcholinesterase Principles of Marigold (Calendula officinalis) Flowers and Preparations
Source: Int J Mol Sci. 2017 Aug 2;18(8):1685. doi: 10.3390/ijms18081685 (PMC5578075; doi:10.3390/ijms18081685)
Supplement: Supplementary file 1 [file ijms-18-01685-s001.pdf]

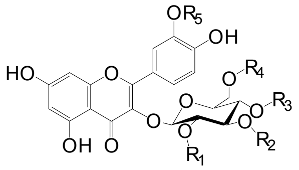

isoquercitrin: $R_1 = R_2 = R_3 = R_4 = R_5 = H$

quercetin-3-*O*-(2″-acetyl)-glycoside: R_1 = Ac, $R_2 = R_3 = R_4 = R_5 = H$

quercetin-3-*O*-(6″-acetyl)-glycoside: $R_1 = R_2 = R_3 = R_5$ = H, R_4 = Ac

quercetin-3-*O*-(2″,6″-di-acetyl)-glycoside: $R_1 = R_4$ =Ac, $R_2 = R_3 = R_5$ = H

calendoflavobioside: R_1 = α-L-Rha*p*, $R_2 = R_3 = R_4 = R_5$ = H

calendoside II: $R_1 = R_3 = R_4 = R_5$ = H, R_2 = α-L-Rha*p*

calendoside I: $R_1 = R_2 = R_4 = R_5$ = H, R_3 = α-L-Rha*p*

rutin: $R_1 = R_2 = R_3 = R_5$ = H, R_4 = α-L-Rha*p*

manghaslin: $R_1 = R_4$ = α-L-Rha*p*, $R_2 = R_3 = R_5$ = H

isorhamnetin-3-*O*-glucoside: $R_1 = R_2 = R_3 = R_4$ = H, R_5 = CH_3

isorhamnetin-3-*O*-(2″-acetyl)-glycoside: R_1 = Ac, $R_2 = R_3 = R_4$ = H, R_5 = CH_3

isorhamnetin-3-*O*-(6″-acetyl)-glycoside: $R_1 = R_2 = R_3$ = H, R_4 = Ac, R_5 = CH_3

isorhamnetin-3-*O*-(2″,6″-di-acetyl)-glycoside: $R_1 = R_4$ =Ac, $R_2 = R_3$ = H, R_5 = CH_3

calendoflavoside: R_1 = α-L-Rha*p*, $R_2 = R_3 = R_4$ = H, R_5 = CH_3

calendoside IV: $R_1 = R_3 = R_4$ = H, R_2 = α-L-Rha*p*, R_5 = CH_3

calendoside III: $R_1 = R_2 = R_4$ = H, R_3 = α-L-Rha*p*, R_5 = CH_3

narcissin: $R_1 = R_2 = R_3$ = H, R_4 = α-L-Rha*p*, R_5 = CH_3

typhaneoside: $R_1 = R_4$ = α-L-Rha*p*, $R_2 = R_3$ = H, R_5 = CH_3

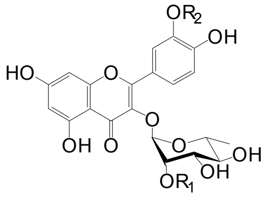

quercitrin: $R_1 = R_2$ = H

quercetin-3-*O*-(2″-ramnosyl)-rhamnoside: R_1 = α-L-Rha*p*, R_2 = H

isorhamnetin-3-*O*-rhamnoside: R_1 = H, R_2 = CH_3

calendoflaside: R_1 = α-L-Rha*p*, R_2 = CH_3

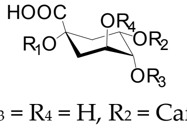

3-*O*-caffeoylquinic acid: $R_1 = R_3 = R_4$ = H, R_2 = CaffA

1,5-di-*O*-caffeoylquinic acid: $R_1 = R_4$ = CaffA, $R_2 = R_3$ = H

3,5-di-*O*-caffeoylquinic acid: $R_1 = R_3$ = H, $R_2 = R_4$ = CaffA

4,5-di-*O*-caffeoylquinic acid: $R_1 = R_2$ = H, $R_3 = R_4$ = CaffA

Figure S1. Structures of compounds from *C. officinalis*. Ac: acetyl; CaffA: caffeoyl and α-l-Rha*p*: α-l-rhamnopyranosyl.
